# Marsh bird occupancy of wetlands managed for waterfowl in the Midwestern USA

**DOI:** 10.1371/journal.pone.0228980

**Published:** 2020-02-21

**Authors:** Therin M. Bradshaw, Abigail G. Blake-Bradshaw, Auriel M. V. Fournier, Joseph D. Lancaster, John O’Connell, Christopher N. Jacques, Michael W. Eichholz, Heath M. Hagy

**Affiliations:** 1 Department of Biological Sciences, Western Illinois University, Macomb, Illinois, United States of America; 2 Prairie Research Institute, University of Illinois at Urbana Champaign, Havana, Illinois, United States of America; 3 Stephen A. Forbes Biological Station, Illinois Natural History Survey, University of Illinois at Urbana-Champaign, Havana, Illinois, United States of America; 4 Cooperative Wildlife Research Laboratory, Center for Ecology, Department of Zoology, Southern Illinois University, Carbondale, Illinois, United States of America; University of Central Florida, UNITED STATES

## Abstract

Marsh birds (rallids, bitterns, and grebes) depend on emergent wetlands, and habitat loss and degradation are the primary suspected causes for population declines among many marsh bird species. We evaluated the effect of natural wetland characteristics, wetland management practices, and surrounding landscape characteristics on marsh bird occupancy in Illinois during late spring and early summer 2015–2017. We conducted call-back surveys following the North American Standardized Marsh Bird Survey Protocol three times annually at all sites (2015 *n = 49*, 2016 *n = 57*, 2017 *n = 55*). Across all species and groups, detection probability declined 7.1% ± 2.1 each week during the marsh bird survey period. Wetlands managed for waterfowl (ducks, geese, and swans) had greater occupancy than reference wetlands. Marsh bird occupancy increased with greater wetland complexity, intermediate levels of waterfowl management intensity, greater proportions of surface water inundation, and greater proportions of persistent emergent vegetation cover. Wetland management practices that retain surface water during the growing season, encourage perennial emergent plants (e.g., *Typha* sp.), and increase wetland complexity could be used to provide habitat suitable for waterfowl and marsh birds.

## Introduction

Greater than 50% of wetlands across the United States were drained and converted to alternate land uses by the 1970s [1–3]. Following that great loss and degradation of wetlands, there is increasing pressure on extant wetlands, some of which are traditionally managed for migrating and wintering waterfowl (ducks, geese and swans), to support the full suite of wetland dependent species [4–6]. Multi-species management of existing wetlands may be needed to sustain or increase wetland bird populations if widespread wetland creation and restoration is not practical [7, 8].

Marsh birds (rallids, bitterns and grebes) are a group of wetland-dependent migratory birds associated with emergent vegetation communities (i.e., persistent and non-persistent emergent vegetation) and often characterized by their elusive nature. Most marsh bird species in North America have experienced population declines primarily due to wetland loss and degradation [7, 9–12]. In response to population declines, several species of marsh birds have been listed as species of conservation concern at the state, provincial and regional levels [13, 14]. Suitable habitat resources for migrating and breeding marsh birds is present on less than 15% of remaining wetlands [15]. Marsh birds are valuable indicators of wetland condition due to their selection of particular vegetation communities and vulnerability to accumulation of environmental contaminants [16, 17]. Several species also are game species in many US states and Canadian provinces, making the study of their population status and trends of special management concern.

Many factors affect breeding marsh bird abundance and diversity in wetlands, including wetland size and isolation [18–23] and surrounding anthropogenic land use [24]. Although several studies have documented local-scale effects on breeding marsh birds, such as water-vegetation interspersion [6, 25] and vegetation density and height [7, 25, 26], intrinsic vegetation characteristics may be less important than wetland size and surroundings [18, 27]. This complexity in marsh bird response to wetland landscape position and vegetation leads to questions about how wetland management impacts the vegetation community and structure.

In wetland management by many state and federal agencies, non-profit conservation organization, and private individuals, hydrology often is manipulated to promote early-successional vegetation (e.g., moist-soil management) that provides food and habitat for waterfowl during the non-breeding season [28, 29]. These managed wetlands often undergo dewatering and soil disturbance in spring and early summer which may have negative effects on breeding or migrating marsh birds through direct mortality, increased predation, or making habitat unavailable [30]. Although multiple studies suggest active wetland management practices may positively influence marsh bird occupancy, it is unknown how the intensity and timing of management practices impact breeding marsh birds [7, 31]. Many wetland conservation and restoration initiatives encourage multi-species design and management, yet waterfowl often are the primary focal group. Limited research is available to indicate how waterfowl management practices affect other migratory bird species [4, 32, 33].

Our goal was to estimate marsh bird occupancy across a range of wetland vegetation communities and management practices in Illinois, USA during late spring and early summer 2015–2017. Our primary study objectives were to assess wetland occupancy by marsh birds relative to 1) local scale characteristics; 2) landscape scale characteristics; 3) management practices for waterfowl and other migratory wetland birds; and 4) stressors related to human activities both within and surrounding the wetlands. We predicted that marsh bird occupancy would increase with coverage of emergent vegetation, and marsh bird occupancy would be greater in wetlands managed for waterfowl than unmanaged wetlands.

## Methods

All field work was done under special use permits from the appropriate state and federal agencies and was conducted in strict accordance with the protocol approved by the Institutional Animal Care and Use Committee at the University of Illinois (approval number 15029). We monitored marsh birds on public and private wetlands across Illinois during 2015–2017. Each wetland was categorized as either a wetland managed for waterfowl or a reference wetland. Wetlands managed for waterfowl are typically impounded on one or more sides by levees and have water control structures allowing hydrological manipulation, including the purposeful drawdown of water to expose soil during the growing season and promote early-successional, annual seed producing plants desirable for waterfowl [34]. Water drawdowns typically occur in late spring or early to mid-summer to provide a suitable window for vegetation to mature and produce seed by autumn, and vegetation is reflooded in autumn to make seed available to migrating and wintering waterfowl [35]. We assembled a comprehensive sampling frame of wetlands managed for waterfowl and other wetland-dependent migratory birds (e.g., secretive marsh birds) within Illinois using previous studies [e.g., 36, 37] and correspondence with Illinois Department of Natural Resources site managers and biologists, private landowners, and Illinois Natural History Survey staff. We defined waterfowl management to include manipulation of vegetation, hydrology, and/or soils (i.e., disking, planting, drawdowns) with the intent of increasing food production or habitat suitability for waterfowl and other wetland-dependent migratory birds in the previous growing season [35]. We randomly selected 20 wetlands managed for waterfowl for sampling each year. A subset of eight sites managed for waterfowl were visited multiple years due to a limited number of wetlands with the breadth of desired conditions for marsh birds (i.e., presence of dense, emergent vegetation).

**Fig 1:**
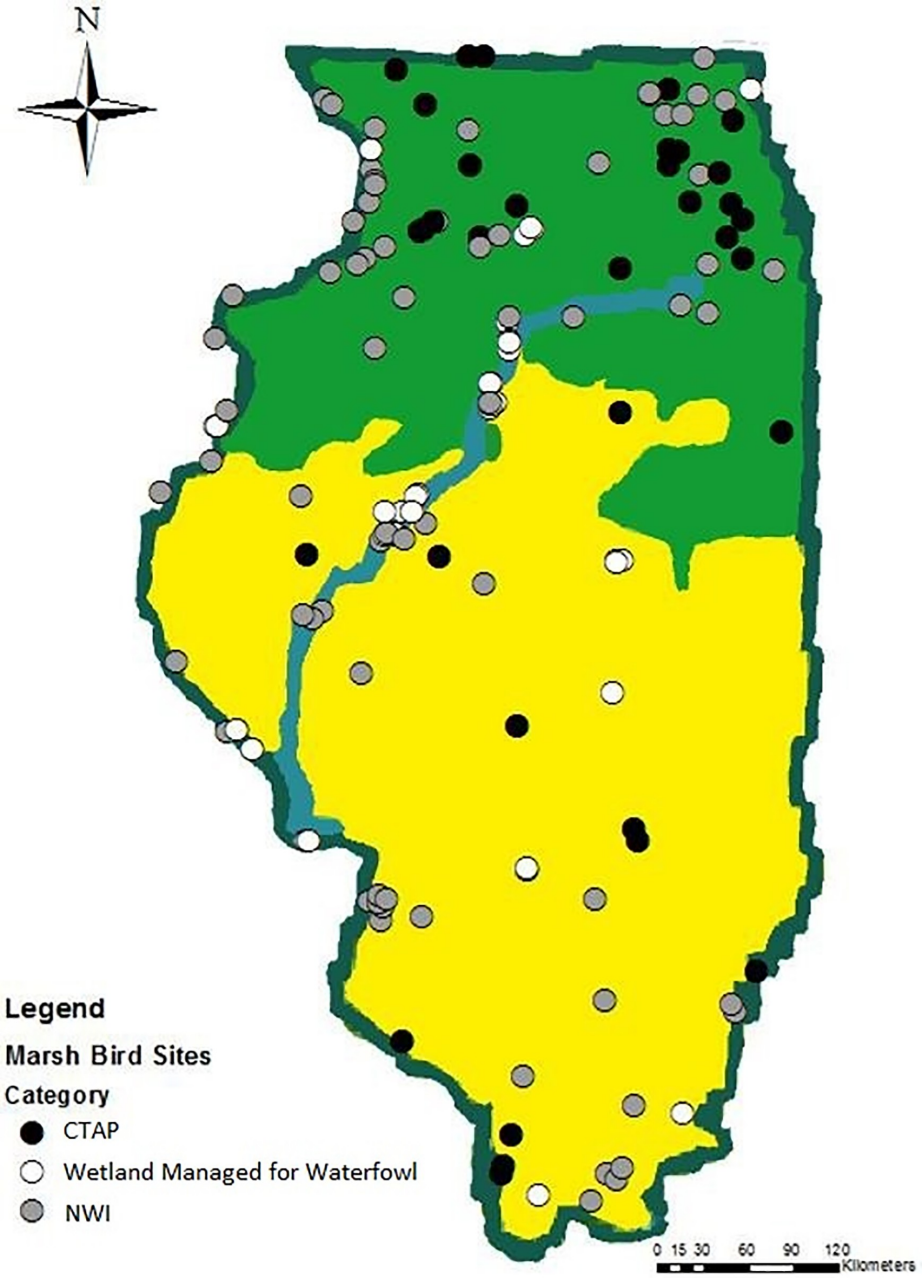
Marsh bird survey sites for two regions of Illinois categorized by average maximum temperatures in May from the PRISM Climate Group at Oregon State University (Conway 2011). Sites consisting of National Wetland Inventory (NWI; grey), wetlands managed for waterfowl (white), and Critical Trends Assessment Program (CTAP; black) wetlands.

Reference wetland categories were created from the National Wetland Inventory (NWI) and Illinois Critical Trends Assessment Program (CTAP) to meet objectives of several joint research projects. We selected potential sample sites for NWI wetlands using a stratified random sampling design. We stratified Illinois by natural division and used a modification of Neyman allocation to weight the number of potential sites per division; total wetland area and variation in wetland density were used for the allocation [38]. The Lake Michigan division and any wetlands <0.5 ha were excluded for logistical reasons. Potential survey sites were then assigned from the NWI using the Create Spatially Balanced Points tool in ArcGIS 10.3, which is an implementation of the reverse randomized quadrant-recursive raster algorithm [39,40]. We used standardized 10-m wetland density as the probability raster for the algorithm. We consolidated NWI polygons into six types/categories (Freshwater Pond, Lake, Freshwater Emergent [herbaceous only], Freshwater Scrub-Shrub/Forested, Riverine, and Other). From 60 CTAP wetlands already scheduled for sampling by collaborators, we used aerial imagery to subset all wetlands with probable habitat suitability for marsh birds (presence of emergent wetland habitat) [6]. We randomly selected 20 wetlands from each respective wetland management category sampling pool, resulting in 60 total sites (Fig 1). If any site was deemed unsuitable for marsh birds during the first visit (i.e., lacking inundation or emergent vegetation) we replaced it with another randomly selected site from the respective sample population.

### Marsh bird surveys

All sample points were located in areas that were within or adjacent to emergent aquatic vegetation (e.g., *Typha* sp) and spaced ≥ 400 m apart to reduce the chances of double counting individuals [9,41]. We established fixed sample points (*n* = 1–5) at each selected site with the number of points allocated to each wetland proportional to overall size and shape, while maintaining 400 m spacing. We restricted the maximum number of survey points to five per site to allow observers to survey multiple wetlands in a single day.

We surveyed for marsh birds following the North American Standardized Marsh Bird Survey Protocol (NASMBSP) [9], which incorporates a repeated call-broadcast survey design. Surveys encompassed the 100-m-radius circle from the marked point. We surveyed each point three times, once each in three separate two-week survey periods from 2015–2017. Illinois encompasses two NASMBSP survey zones (Fig 1; [9]), so surveys began two-weeks later in the northern half of the state (i.e., southern zone start date = 15 April, northern zone start date = 1 May, survey window was 6 weeks long in both zones). We conducted all surveys between one-half hour before sunrise and approximately 2 hours after sunrise and avoided heavy rains or high wind conditions to maximize detection probability [9].

Following the NASMBSP, we used a 5-min passive survey and subsequent 1-min alternating series of 30 sec periods of calls and silence in the following fixed order, which progressed from least to most intrusive species: black rail (*Laterallus jamaicensis*), least bittern (*Ixobrychus exilis*), yellow rail (*Coturnicops noveboracensis*), sora (*Porzana carolina*), Virginia rail (*Rallus limicola*), king rail (*Rallus elegans*), American bittern (*Botaurus lentiginosus*), common gallinule (*Gallinula galeata*), American coot (*Fulica americana*), and pied-billed grebe (*Podilymbus podiceps*; [9] We selected these marsh bird species because they have part of their potential breeding range in Illinois. We broadcasted calls using electronic game callers (Western Rivers Pursuit, Maestro Game Calls, LLC., Dallas, Texas, USA; or Primos Turbo Dogg, Primos Hunting, Flora, Mississippi, USA). Calls were broadcasted at a volume of >80 dB with the observer positioned ~1 m from the game caller [9]. To account for variation in detection probability, we also recorded variables including wind speed using the Beaufort scale (values 0–5), temperature (° C), cloud cover representing severity of weather (values 0–7), background noise intensity (values 0–4), and the name of the observer [9].

### Wetland conditions

**Fig 2:**
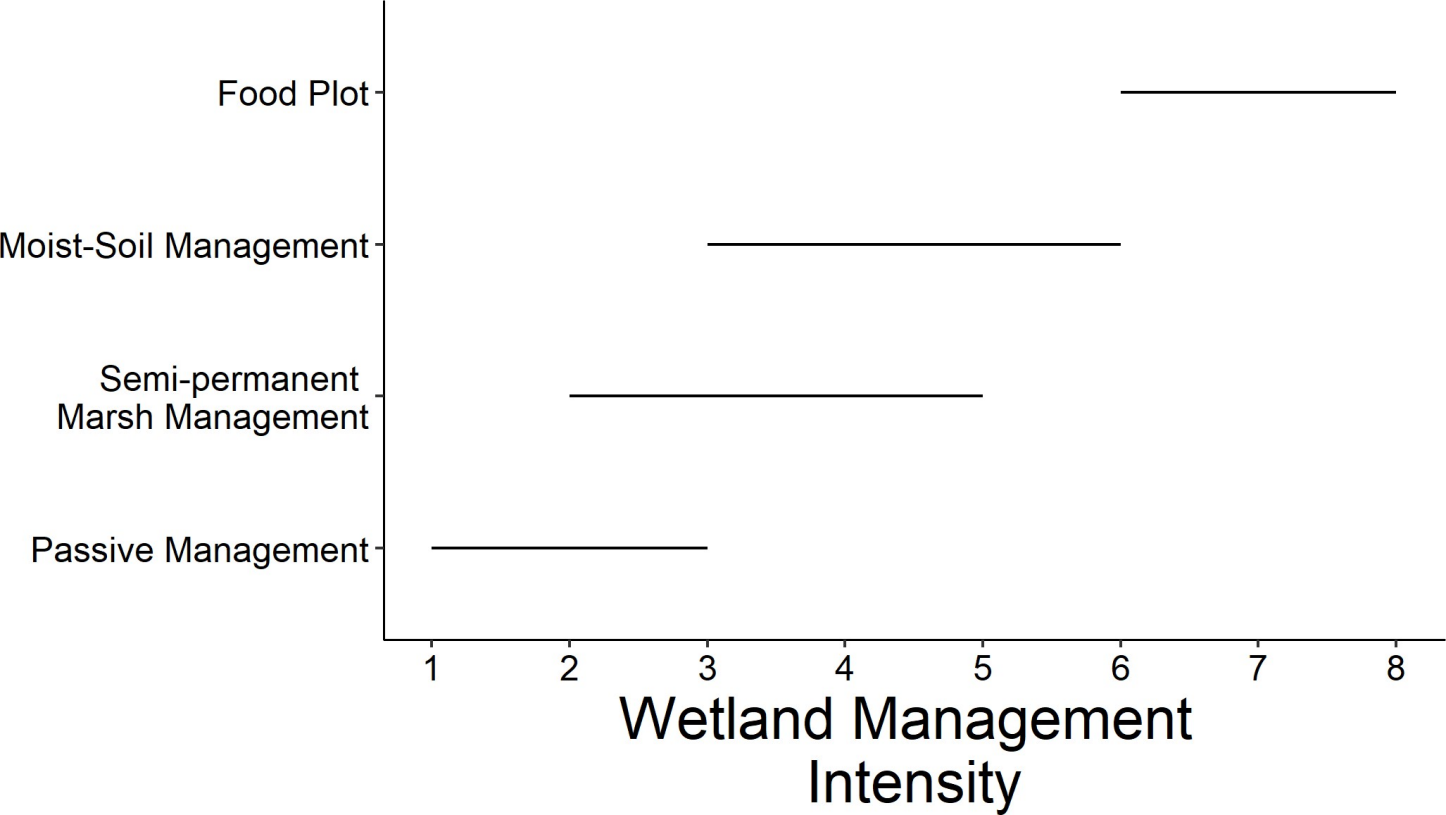
Example wetland management regimes (Y axis) and the range of waterfowl management intensity values (X axis) typically encountered based a visual index of management activities and capabilities present (1 [low intensity or passive management]– 8 [intensive/active management]).

We evaluated wetland conditions at each sample point and across the entire site (Table 1). At the site level, we assessed four variables: the intensity of waterfowl management activities, wetland complexity, wetland connectivity and anthropogenic disturbance. These four variables were assessed in person by conducting visual assessments from representative vantage points throughout the site and served as an index across all sample points and sites in our study area. A visual index of waterfowl management intensity in a given wetland was categorized on an eight-point scale based on evidence of management activities from the previous growing season and presence of infrastructure to facilitate water and vegetation management for waterfowl (1 [no waterfowl management; e.g., lack of evidence of managed drawdowns, vegetation manipulation, or water control ability]– 8 [very intense waterfowl management and presence of levees, pumps, and water control structures; e.g., annual soil disturbance, disking and planting food plots, etc.]; Fig 2). Wetland cover type complexity was indexed on a six-point scale (1 [homogeneous]– 6 [high heterogeneity]). Wetland connectivity in relation to other wetlands was indexed on an eight-point scale (1 [isolated from other wetlands]– 8 [adjacent and connected to other wetlands]). Anthropogenic disturbance was measured using a modification of the Ohio Rapid Assessment Method (ORAM), which includes potential stressors and indicators of wetland condition, including metrics indicative of wetland quality for marsh birds under a wide variety of modified conditions specific to the Midwest region (e.g., management of hydrology, presence of water control structures, drawdown timing, urban development and adjacent agricultural land use; [15]). Across the site, we also recorded average water depth by wading into wetlands at numerous points, categorized into four depth ranges (1: dry; 2: very shallow, <10 cm; 3: shallow, <45 cm; 4: deep, >45 cm) and recorded percent surface-water inundation [2, 9] by visual estimation. At each sample point (a 100 meters diameter circle), we assessed percent cover by vegetation type (dense persistent emergent [hereafter, persistent emergent], non-persistent emergent, scrub-shrub, forested, non-rooted floating aquatic vegetation, open water, and aquatic bed i.e., floating-leaf and submersed aquatic vegetation).

**Table 1:** List of factors and the corresponding units used as possible predictors of marsh bird detection and abundance in Illinois during late spring and early summer 2015–2017.

Model Group	Factors	Type and Units/Scale/Range
Detection	Time Relative to Sunrise	Continuous: Minutes
	Temperature	Continuous: Degrees Celsius
	Sky Cover	Ordinal: 0–8
	Wind	Ordinal: 0–5
	Background Noise	Ordinal: 0–4
	Observer (s)	Categorical: Observer
	Year	Continuous: 2015–2017
Occupancy	Waterfowl Management Intensity	Ordinal: 1–8
	Wetland Complexity	Ordinal: 1–6
	Connectivity to Rivers or Streams	Ordinal: 0–7
	Management Category	Categorical: Unmanaged, Passive, Active
	Survey Period	Categorical: 1, 2, 3
	Survey Region	Categorical: North or South
	Wildlife Management Intensity	Ordinal: 0–7
	Site Type	Categorical: Critical Trends Assessment Program (CTAP), National Wetland Inventory (NWI), Managed for Waterfowl
	Water Depth	Ordinal: 0–4
	Surface Water Inundation	Continuous: % of Survey Point
	Aquatic Bed	Continuous: % of Survey Point
	Dense Persistent Emergent Vegetation	Continuous: % of Survey Point
	Non-persistent Emergent Vegetation	Continuous: % of Survey Point
	Shrub-Scrub	Continuous: % of Survey Point
	Forested	Continuous: % of Survey Point
	Open Water	Continuous: % of Survey Point
	Natural Division	Categorical Variable
	ORAM Factors	Categorical Variable

### Data analyses

We estimated occupancy and detection probability of marsh birds across sites using the occu() function in the *unmarked* package for program R, version 3.1 [42,43]. An important assumption regarding detection probability from repeated surveys is that the population is closed: that is, no immigration or emigration of individuals among sampling periods [44]. Violating this assumption can lead to underestimating detection probability and overestimating occupancy. For example, inclusion of migrating individuals would violate the assumption of a closed population across the six-week survey period and negatively bias estimates of detection probability. We judged that our data were unlikely to meet the closure assumption based on anecdotal observations from the field and raw detection rates (proportion of sites with a detection during a given visit) which declined with each repeated visit. Past research suggests that marsh bird migration continues throughout the monitoring season outlined in the NASMBSP [45]. Calculating occupancy for mobile or migrating organisms may require shorter periods between repeat visits or the use of spatial replication across multiple surveys within the same area of study [46,47]. Thus, we estimated detection probability among sample points within each site and survey period instead of across survey periods. Under this design, we assumed that if a species was present at a single point within a site, it was present at all points within that site, and non-detection was a false negative. Due to similarities in vegetation and wetland characteristics among survey points within each site and distribution of sample sites in relation to mostly unsuitable habitat surrounding sites, we believe this approach was reasonable and that the probability of meeting this assumption was substantially greater than that of population closure across the six-week survey period [46].

Small sample sizes for most individual species precluded species-specific estimation of occupancy, so we grouped species with similar habitat requirements and relevance to management [31]. Our three groups included a group with all marsh bird species (hereafter the comprehensive group), one with species associated with emergent vegetation (least bittern, American bittern, king rail, sora, Virginia rail, and yellow rail), and one with species associated with open water (American coot, common gallinule, and pied-billed grebe; [31]).

We used a two-step modeling process by which covariates for detection (*p*) were modeled first while keeping occupancy (Ψ) constant at null. We then used the highest-ranked model for detection in all subsequent models for occupancy [2,48]. We assessed correlation among the site-specific covariates by constructing a correlation matrix prior to analysis and removing one of each of the correlated variables (*|r|* > 0.5; [2]). We retained variables that were most relevant to wetland management and most biologically plausible to influence detection. We modeled occupancy as a function of all remaining independent variables individually and then built additive models using biologically plausible combinations of variables that received the most support [2]. We compared candidate models using Akaike’s Information Criterion (AIC) and considered models ΔAIC ≤ 2 to be competitive, and models ΔAIC >2 to be non-competitive [49]. We then used odds ratios to illustrate effect sizes of variables included in all competitive models [50]. We determined associations between response and predictor variables using odds ratios. The odds ratio for a predictor variable is the relative amount by which the odds of the outcome increase (odds ratio >1.0) or decrease (odds ratio <1.0) with each unit increase in the predictor variable [50]. As a result, each odds ratio approximates the likelihood of a predicted outcome among associated variables.

## Results

**Fig 3:**
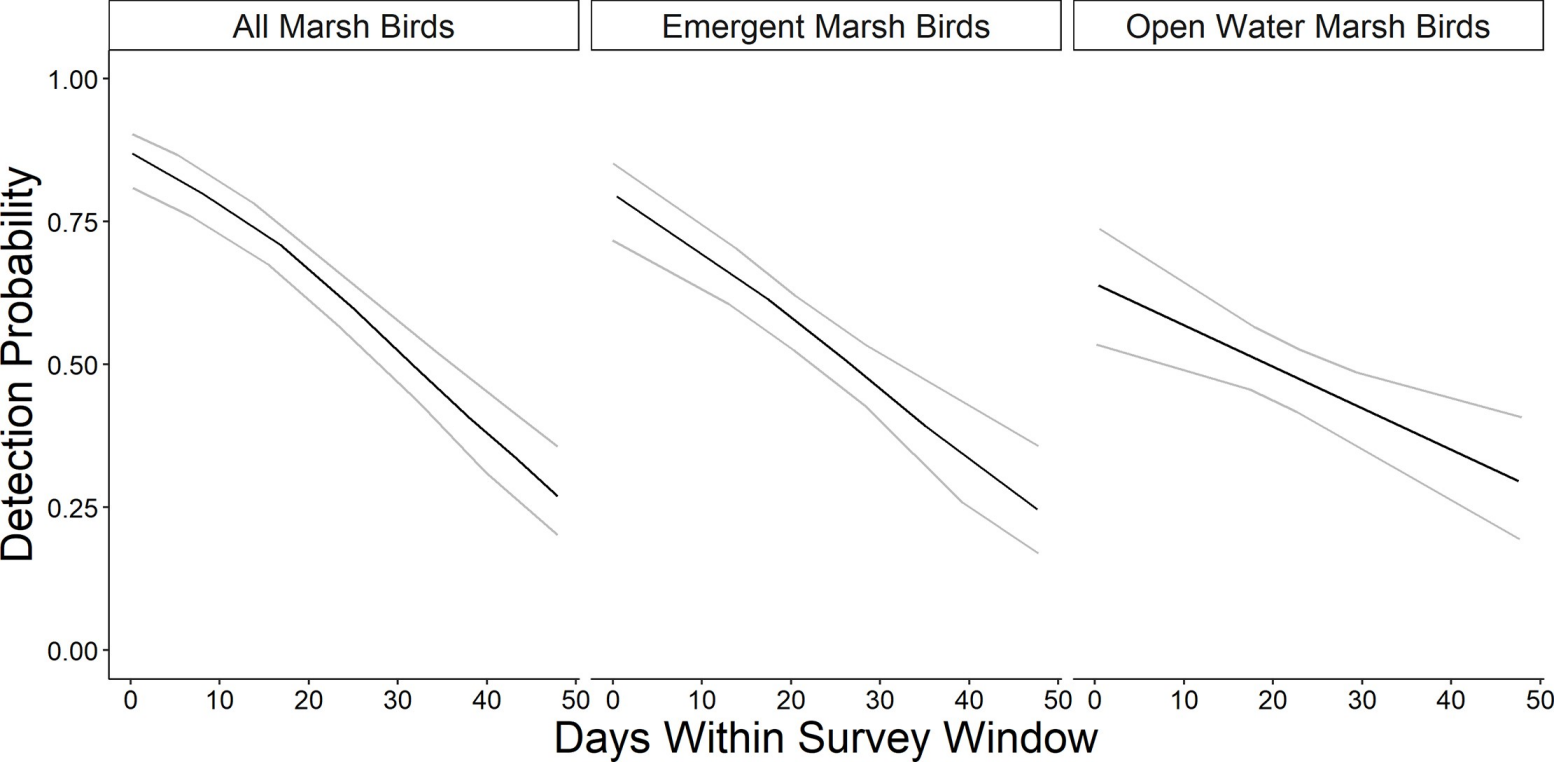
Model-estimated marsh bird detection probability (black line) for all three marsh bird groups (± 95% confidence limits [grey lines]) by adjusted date. Surveys were conducted from day 0 (April 15 or May 1, depending on latitude stratification) to day 48 across Illinois during late spring and early summer 2015–2017.

We recorded 3,680 marsh bird detections during 1,033 surveys at 380 sample points between 2015–2017 (For site locations see S1 Appendix). Due to differences in wetland size and area of emergent vegetation potentially suitable for marsh bird occupancy, we sampled 1.4 points per CTAP wetland (SD = 0.7), 2.1 points per NWI wetland (SD = 1.2) and 3.5 points per wetland managed for waterfowl (SD = 1.5). American coot were most commonly detected (61.3%), followed by sora (26.7%), pied-billed grebe (5.5%), common gallinule (2.5%), Virginia rail (1.5%), least bittern (1.4%), American bittern (0.9%), king rail (0.2%), and yellow rail (0.1%). We detected no black rail during our surveys. Ordinal date was the highest-ranked model for detection probability for all three marsh bird groups (Table 2). The probability of detection declined 0.06 per day (odds ratio = 0.94; 95% CI = 0.93–0.96) for the comprehensive group, 0.05 per day (odds ratio = 0.95; 95% CI = 0.93–0.97) for emergent vegetation group, and 0.03 per day (odds ratio = 0.97; 95% CI = 0.95–0.99) for the open-water group (Fig 3).

**Table 2:** Model rankings for variables predicting detection probability by species groupings of marsh birds based on Akaike’s Information Criterion (AIC), difference in AIC relative to the highest-ranked model (ΔAIC), relative model weight (*w_i_*), and number of parameters (*K*) from surveys conducted at wetlands throughout Illinois during late spring and early summer 2015–2017.

Bird Group	Model^a^	ΔAIC	*w*_*i*_	*K*
Comprehensive	ordinal date	1276.99	0.00	1.00	3
	temperature	1324.58	47.59	0.00	3
	sky cover	1330.07	53.08	0.00	8
Emergent^b^	ordinal date	1200.32	2.78	0.20	3
	temperature	1249.32	51.78	0.00	3
	year	1252.63	55.09	0.00	3
Open^c^	ordinal date	1008.4	1.36	0.13	3
	time^d^	1020.43	13.39	0.02	3
	null	1020.65	13.61	0.00	2

^a^ For all models, the occupancy parameter was held constant.

^b^ Emergent = American bittern, *Botaurus lentiginosus*; least bittern, *Ixobrychus exilis*; sora, *Porzana carolina*; king rail, *Rallus elegans*; Virginia rail, *Rallus limicola*; and yellow rail, *Coturnicops noveboracensis*.

^c^ Open = American coot, *Fulica americana*, common gallinule, *Gallinula galeata*, and pied-billed grebe, *Podilymbus podiceps*.

^d^ Time = time since sunrise.

**Fig 4:**
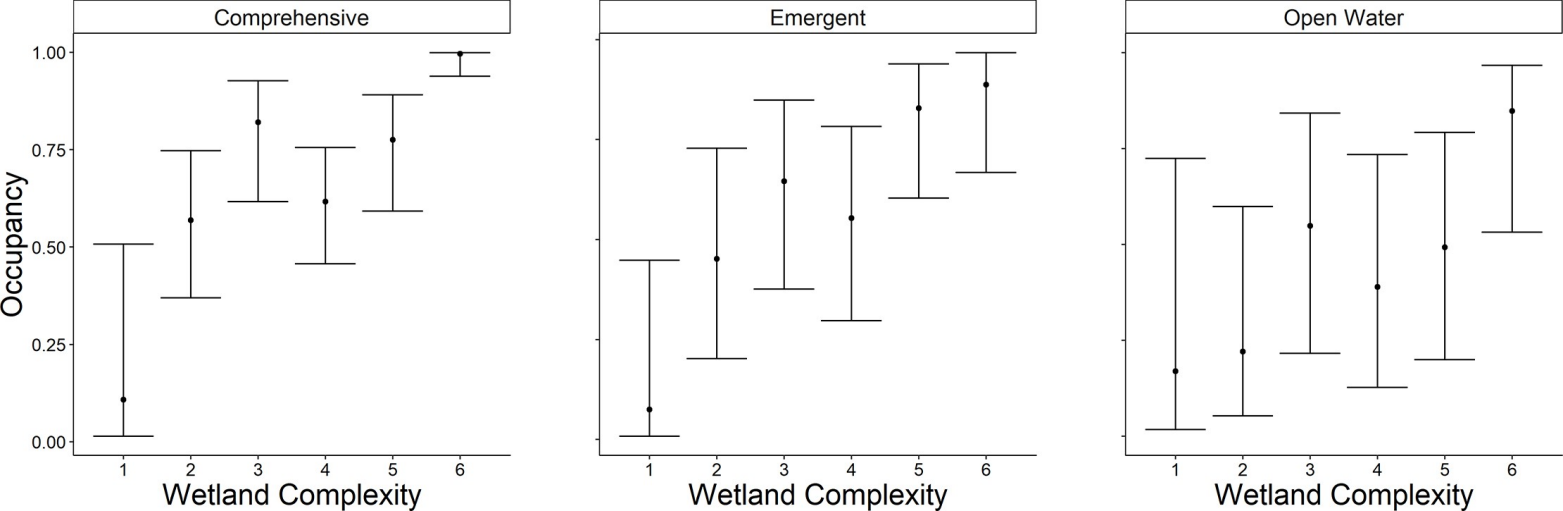
Predicted probability (95% confidence limits) of site occupancy across wetland complexity levels in Illinois during late spring and early summer 2015–2017. Site type (National Wetland Inventory) was held constant. Each box represents one grouping of species, labeled at the top, with comprehensive being all the species in open water and emergent combined.

**Fig 5:**
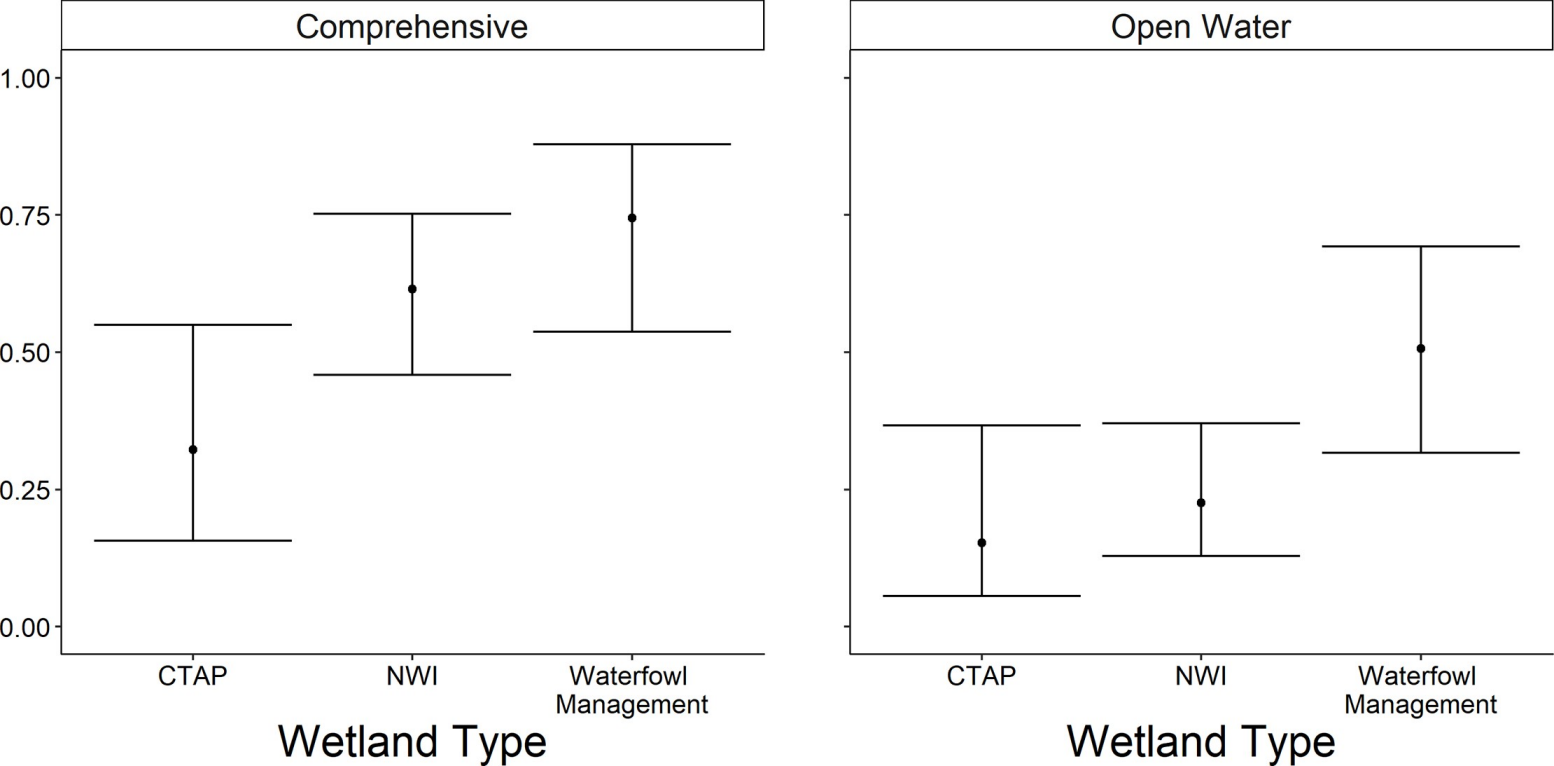
Predicted probability (95% confidence limits) of site occupancy for the comprehensive group across wetlands managed for waterfowl, Critical Trends Assessment Program (CTAP), and National Wetland Inventory (NWI) site types in Illinois during late spring and early summer 2015–2017. Each box represents one grouping of species, labeled at the top, all species in open water are in comprehensive.

The highest-ranked model for occupancy of the comprehensive group included wetland complexity and wetland type; weight of evidence (*w*_*i*_) supporting this model was substantial (*w*_*i*_ = 0.97; Table 3). All other models were considered to not have substantial support (*w*_*i*_ < 0.03) and thus, we excluded them from further consideration. The probability of wetland occupancy was 28.7 times greater (odds ratio = 28.7; 95% CI = 3.1–271.0) at sites with the greatest level of complexity compared to sites with the lowest level of complexity (Fig 4). The probability of occupancy increased 1.80 times (95% CI = 0.7–4.6) between NWI and sites managed for waterfowl (Fig 5). The probability of occupancy decreased 0.71 times (odds ratio = 0.29; 95% CI = 0.31–0.88) between NWI and CTAP sites (Fig 5).

**Table 3:** Model rankings for variables predicting occupancy probability by species groupings of marsh birds based on Akaike’s Information Criterion (AIC), difference in AIC relative to the highest-ranked model (ΔAIC), relative model weight (*w_i_*), and number of parameters (*K*) from surveys conducted at wetlands throughout Illinois during late spring and early summer 2015–2017.

Bird Group	Model^a^,^b^	AIC	ΔAIC	*w*_*i*_	*K*
Comprehensive	CMP + TYP	1268.74	0.00	0.97	10
	CMP	1277.13	8.39	0.02	8
	PIN	1277.39	8.65	0.01	5
	TYP	1299.21	30.47	0.00	10
	NULL	1331.08	62.48	0.00	3
Emergent^c^	CMP + PIN + PDP + PRD	1174.11	0.00	0.80	12
	CMP + PDP + PRD	1177.68	3.57	0.13	11
	CMP + PIN + PRD	1179.27	5.16	0.06	11
	CMP + PRD	1197.39	23.28	0.00	10
	NULL	1265.60	91.49	0.00	3
Open^d^	CMP + TYP + WTR	949.08	0.00	0.90	17
	CMP + TYP	954.22	5.14	0.07	10
	CMP + WTR	956.07	6.99	0.03	15
	TYP + WTR	967.22	18.14	0.00	12
	NULL	1020.65	71.56	0.00	3

^a^ All occupancy models presented contained the variable ordinal date in detection probability

^b^ CMP = wetland complexity, TYP = Site Type, WTR = waterfowl management intensity, PIN = Percent Inundation, PDP = Percent cover dense persistent emergent vegetation, PRD = Survey Period, and NULL = intercept only.

^c^ Emergent = American bittern, *Botaurus lentiginosus*; least bittern, *Ixobrychus exilis*; sora, *Porzana carolina*; king rail, *Rallus elegans*; Virginia rail, *Rallus limicola*; and yellow rail, *Coturnicops noveboracensis*.

^d^ Open = American coot, *Fulica americana*; common gallinule, *Gallinula galeata*; and pied-billed grebe, *Podilymbus podiceps*

**Fig 6:**
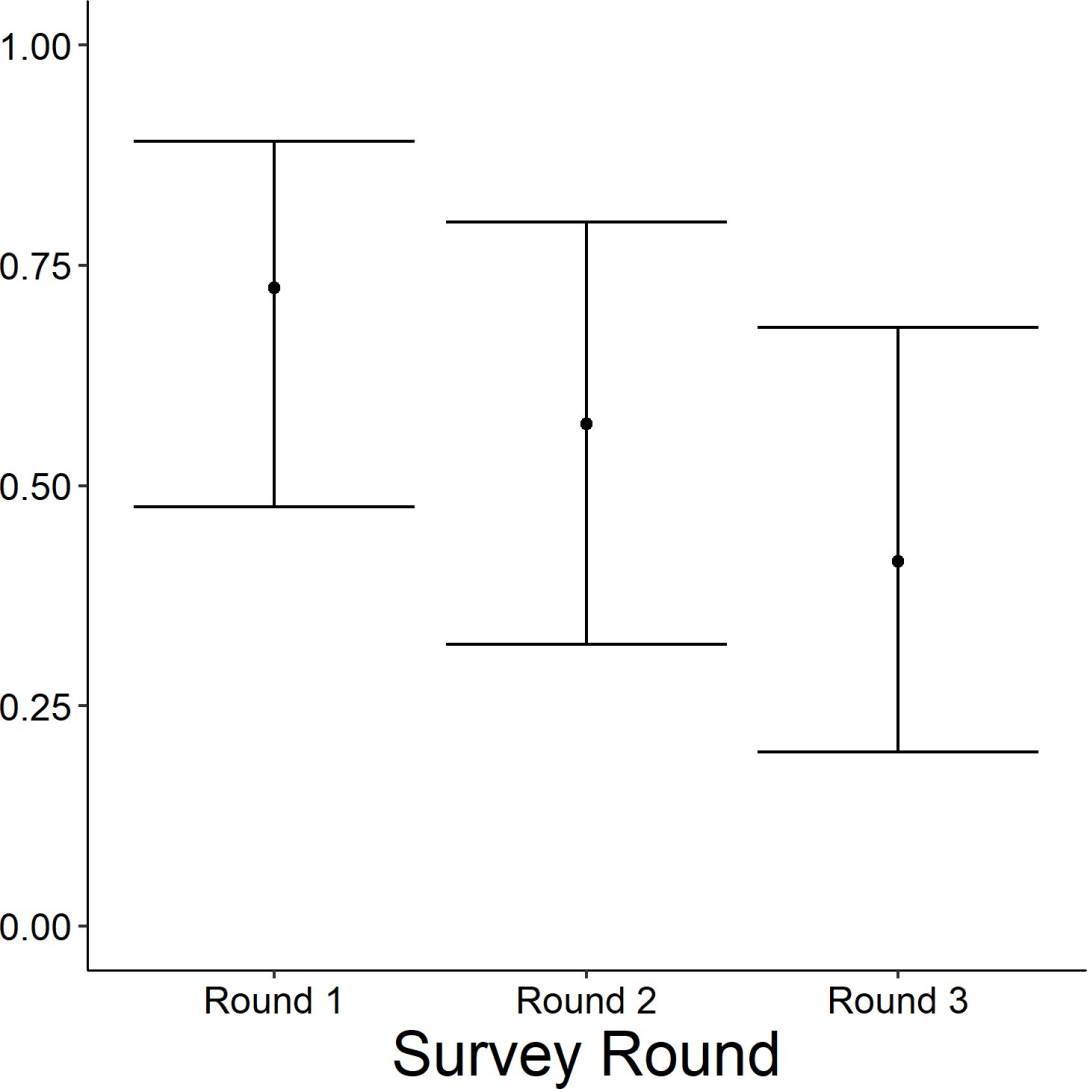
Predicted probability (95% confidence limits) of site occupancy for the emergent marsh bird group (American bittern, *Botaurus lentiginosus*; least bittern, *Ixobrychus exilis*; sora, *Porzana Carolina*; king rail, *Rallus elegans*; virginia rail *Rallus limicola*, and yellow rail, *Coturnicops noveboracensis*) across survey rounds in Illinois during late spring and early summer 2015–2017.

**Fig 7:**
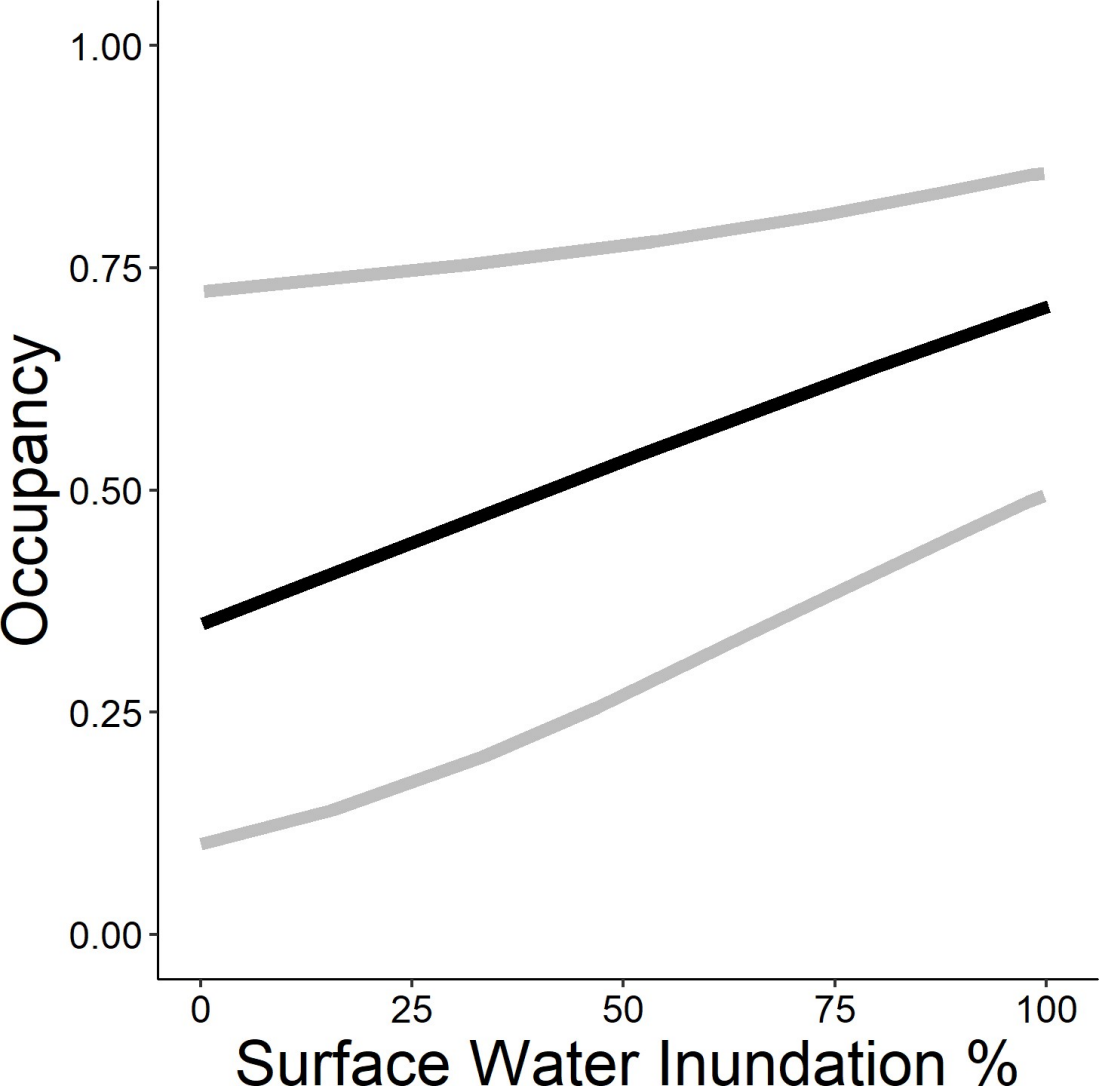
Predicted probability (95% confidence limits) of site occupancy for the emergent marsh bird group (American bittern, *Botaurus lentiginosus*; least bittern, *Ixobrychus exilis*; sora, *Porzana Carolina*; king rail, *Rallus elegans*; virginia rail *Rallus limicola*, and yellow rail, *Coturnicops noveboracensis*) across percent surface water inundation in Illinois during late spring and early summer 2015–2017.

**Fig 8:**
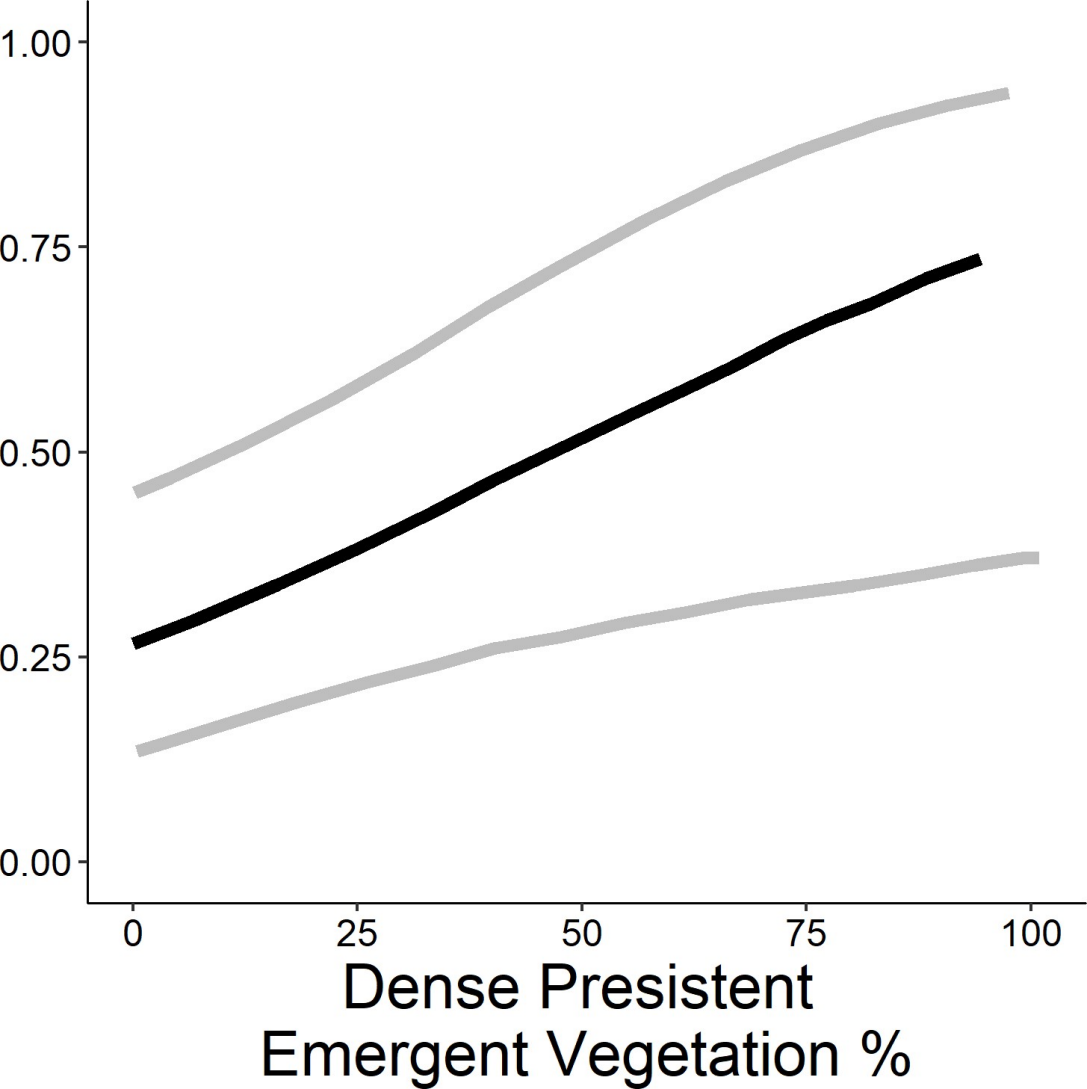
Predicted probability (95% confidence limits) of site occupancy for the emergent marsh bird group (American bittern, *Botaurus lentiginosus*; least bittern, *Ixobrychus exilis*; sora, *Porzana Carolina*; king rail, *Rallus elegans*; virginia rail *Rallus limicola*, and yellow rail, *Coturnicops noveboracensis*) across dense persistent emergent vegetation coverage in Illinois during late spring and early summer 2015–2017.

The highest-ranked model predicting occupancy of the emergent group included wetland complexity, survey period, surface water inundation, and persistent emergent vegetation. Further, the weight of evidence supporting this model was 6.2 times greater (odds ratio = 0.80; 95%; Table 3) than the Wetland Complexity + Percent Cover of Dense Emergent Vegetation + Survey Period model (*w*_*i*_ = 0.13), and 13.3 times greater than the Wetland Complexity + Percent Inundation + Survey Period model (*w*_*i*_ = 0.06). All other models were considered non-competitive (*w*_*i*_ < 0.01) and thus, excluded from further consideration. The probability of occupancy was 0.98 times greater (95% CI = 0.08–1.23) at the highest level of complexity than the lowest (Fig 4). The probability of occupancy decreased 0.49 times (odds ratio = 0.51; 95% CI = -0.86 to 0.23) between survey round 2 and survey round 1, and 0.73 times (95% CI = 0.33–0.89) between survey round 3 and survey round 1 (Fig 6). Probability of occupancy increased 1.02 times (odds ratio = 1.02; 95% CI = 1.00–1.03; Fig 7) for every 1% increase in surface water inundation and 1.03 times (odds ratio = 1.03; 95% CI = 1.00–1.05; Fig 8) for every 1% increase in the percent cover of persistent emergent vegetation.

**Fig 9:**
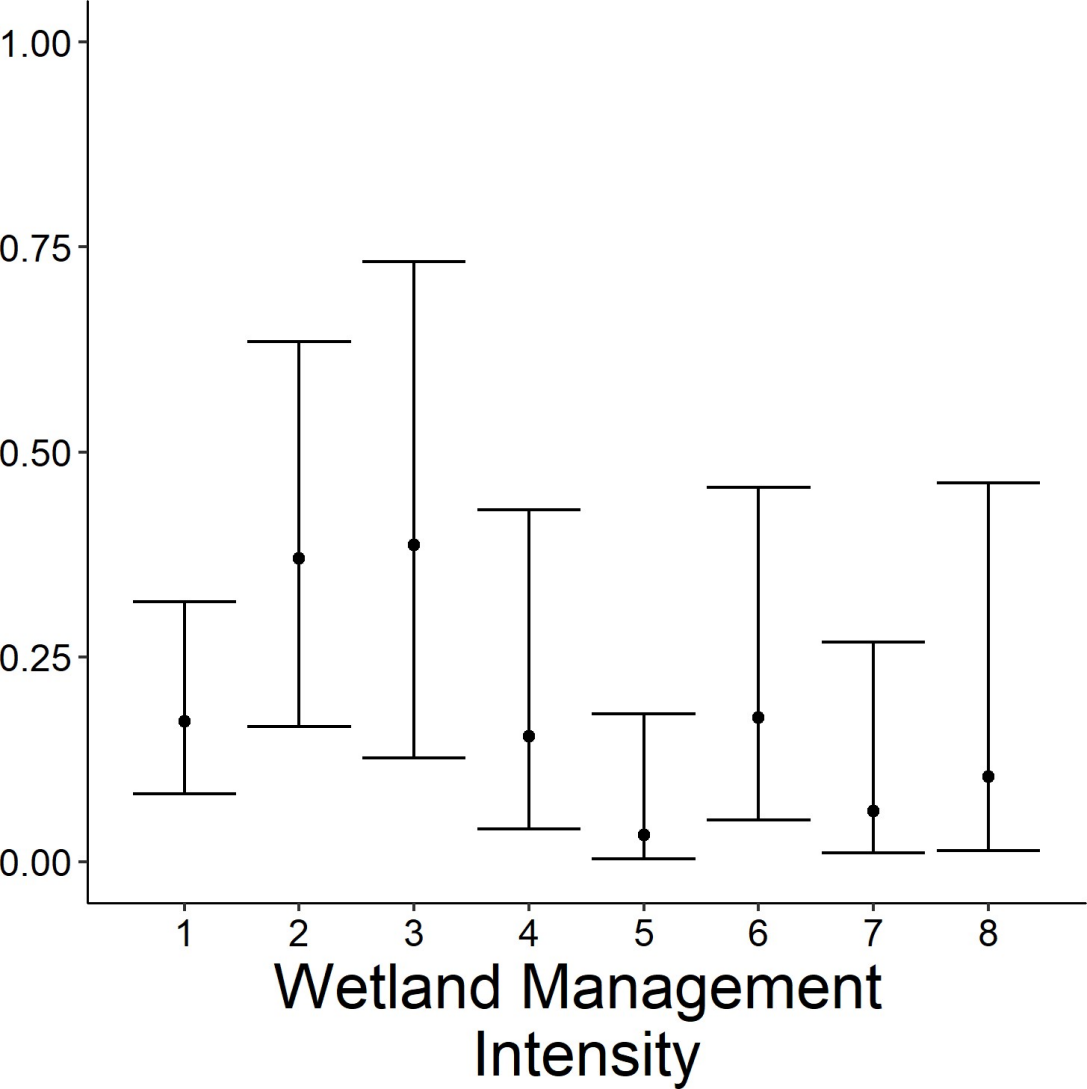
Predicted probability (95% confidence limits) of site occupancy for the emergent marsh bird group (American bittern, *Botaurus lentiginosus*; least bittern, *Ixobrychus exilis*; sora, *Porzana Carolina*; king rail, *Rallus elegans*; virginia rail *Rallus limicola*, and yellow rail, *Coturnicops noveboracensis*) across wetland management intensity in Illinois during late spring and early summer 2015–2017.

The highest-ranked model predicting occupancy of the open-water group included wetland complexity, site type, and waterfowl management intensity; weight of evidence supporting this model was substantial (*w*_*i*_ = 0.90; Table 3). Further, weight of evidence supporting this model was 12.3 times greater than the Wetland Complexity + Site Type model (*w*_*i*_ = 0.07). All other were considered non-competitive (*w*_*i*_ < 0.03) and thus, excluded from further consideration (Table 3). The probability of occupancy increased 28 times (odds ratio = 28; 95% CI = 2–37) between the lowest and highest level of wetland complexity (Fig 4). The probability of occupancy was 5 times greater (odds ratio = 5; 95% CI = 2–12) at wetlands managed for waterfowl compared to NWI wetlands (Fig 5). Moreover, probability of occupancy decreased 0.30 times (odds ratio = 0.70; 95% CI = 0.02–0.77) between NWI wetlands and CTAP wetlands (Fig 5). Occupancy was greatest at intermediate levels of waterfowl management intensity. For instance, the probability of open water-associated marsh birds occupying a wetland was 3 times greater (odds ratio = 3, 95% CI = 1–12) in wetlands with a level 4 management intensity than sites at level 1 management intensity. Furthermore, a level 7 management intensity resulted in a 0.44 times decrease (95% CI = 0.00–0.92) in the probability of occupancy compared to sites at level 1 management intensity (Fig 9).

## Discussion

The conservation community often assumes that wetlands managed for waterfowl provide habitat for other wetland-dependent species, but there are several areas of potential conflict between managing intensively for the dietary needs of waterfowl and providing habitat resources for other wetland dependent birds during the breeding season and migration. Our results indicate that some wetlands managed for waterfowl also support marsh birds, but intensively managed wetlands for waterfowl likely have limited benefit for marsh birds. In particular, our results highlighted the importance of wetland hydrologic and vegetation complexity to breeding marsh birds, which is sometimes discouraged in active wetland management for waterfowl. For example, dense and persistent emergent vegetation (e.g., *Typha* spp.) is often discouraged through hydrological manipulation (e.g., annual drawdowns), chemical control, or physical manipulations to favor annual plants that produce more food for waterfowl. Intensive management that includes early and lengthy drawdowns (April–May through October–November) or exclusion of perennial emergent vegetation limits available migration and breeding habitat for marsh birds [2,30,36,52]. Managed wetlands with later drawdowns (e.g., June–July) and those with less frequent drawdown schedules (e.g., 1 in 3–5 years) have greater potential for marsh bird occupancy, but more work is needed to understand the impacts of management on nest success and survival of marsh bird species throughout the annual cycle [40,53,54]. Our work reinforces previous findings that wetlands with greater hydrological and vegetation complexity that produces dense, emergent vegetation support greater marsh bird occupancy in the Midwest [6,55–59].

Previous work on breeding marsh bird habitat has focused on two scales—the surrounding landscape and local/point scale. Our modified ORAM score accounted for many variables related to the surrounding landscape, including anthropogenic activity and wetland connectivity to other areas, but we did not find differences for any of our marsh bird groups. This finding differs from previous work indicating ORAM score, wetland size, isolation, connectivity, and neighboring land use influenced marsh bird occupancy [18–24,60]. We suspect that high wetland loss and degradation rates have limited suitable marsh bird habitat throughout most of our study area to the point that the presence of dense, emergent vegetation is likely more influential than the surrounding landscape. Several previous studies also noted marsh bird associations with local or point level variables, noting positive effects of interspersion or wetland complexity on marsh bird occupancy [6,7,25]. Wetland vegetation complexity likely provides important habitat edges along which adults may forage while still maintaining nearby cover for themselves and their young.

Non-detection of marsh birds results from a combination of true absence and non-detection. Non-detections can occur when the bird does not call or is not visible during the survey period. Alternatively, non-detections can occur when the observer fails to hear or see the bird despite a response (false absences). These non-detections contrast with true absences when there are no birds there to detect [61]. To overcome these issues, an unbiased estimate of detection probability requires survey replication while the population is closed [44]. There are two primary ways of replicating surveys, over time and over space[25,30,31]. Conway [9] recommended that initial surveys be conducted after migration and before the initiation of breeding, often outlined as a six week window that varies by latitude. If individuals continue to migrate through the site during the survey periods, this can bias occupancy high. We think this is happened on our sites because the majority of our detections occurred in the first and second survey period. The declining trend in detections over a spring migration/early breeding season time period has been documented in other studies [6]. An additional complication is each species has a different migratory and breeding season peak, and so the survey window is likely under sampling some species and oversampling others [40]. Variation in detection could be caused by a wide range of factors, including the physical environment (temperature, precipitation, vegetation structure, wind conditions), behavior (changes in vocalization rate during the migratory period, variation in vocalization rate between birds on territory versus migratory individuals, as well as decreases in vocalizations as the breeding season progresses) and interactions between individuals among others [40], but information about how these factors impact detection or assumptions of closure needs further study. Kaufmann [62] observed that marsh birds gave similar vocalizations during migration and breeding periods. Anecdotal evidence from our study suggests lower occupancy rates during later survey periods for most species. Reducing the time between surveys could decrease the probability of marsh birds moving in or out of the survey area, although more work is needed in this area to ensure that our survey and analytical methods are estimating density in a biologically relevant way during migration.

Wetland management is necessary to provide suitable hydrological and vegetative conditions for migratory waterbirds given the limited quantity and degradation of many remaining wetlands across the Midwestern U.S. Several alternative practices under the broader umbrella of wetland management for waterfowl may be considered by wetland managers to increase occupancy of marsh birds while minimizing potential benefits to waterfowl. When drawdowns and vegetation manipulation is needed to facilitate infrastructure maintenance or modify the vegetation community, managers should consider delaying these practices to maintain hydrology throughout the marsh bird migration period (e.g., April–May). In addition, managers may take advantage of topographical differences within natural wetlands or impoundments to optimize hydrological and vegetation conditions to create vegetation complexity and maintain areas with dense, persistent emergent vegetation flooded during spring and early summer (April–June). Managers that work with a set of individual wetland units or impoundments can manage those as a complex, creating a mosaic of emergent wetland habitat conditions, each meeting specific needs of targeted waterbird guilds.

Increased marsh bird use of wetlands can be achieved within a mosaic of wetland conditions across a wetland complex, by managing a subset of units to increase coverage by dense, persistent emergent vegetation by maintaining surface water during the growing season for several consecutive years. Wetlands with dense emergent vegetation will eventually need to have succession reset, and this can be done as a part of multi-year hydrology management strategies while also providing waterfowl habitat in unity with regional waterfowl management plans [55,57,58]. Creating or managing for emergent marshes with semi-permanent water regimes that provide a mosaic of emergent vegetation, open water, and submersed aquatic vegetation can provide abundant food for waterfowl while promoting use by marsh birds [55, 58]

## Supporting information

S1 Appendix Locations of surveyed wetlands throughout Illinois. (PDF)
