# Supplementary material for: Marsh bird occupancy of wetlands managed for waterfowl in the Midwestern USA
Source: PLoS One. 2020 Feb 21;15(2):e0228980. doi: 10.1371/journal.pone.0228980 (PMC7034909; doi:10.1371/journal.pone.0228980)
Supplement: S1 Appendix — (PDF) [file pone.0228980.s001.pdf]

S1 APPENDIX A.

LOCATIONS OF SURVEYED WETLANDS THROUGHOUT ILLINOIS

Latitude and Longitude of sites surveyed for marsh birds during late spring and early summer 2015–2017 in Illinois. Sites included wetlands managed primarily for migrating waterfowl (i.e., focal) and randomly-selected reference wetlands statewide. Reference wetlands included emergent polygons from the National Wetland Inventory (NWI) and sites from the Illinois Natural History Survey's Critical Trends Assessment Program (CTAP). Region was determined using the North American Secretive Marsh Birds Survey Protocol (NASMBSP; Conway 2011).

Site Number	Year	Category	Region	Longitude	Latitude
1382	2015	CTAP	North	-89.8329078	41.71239348
1402	2015	CTAP	North	-89.85194364	41.70699541
1402	2015	CTAP	North	-89.77166035	41.75011327
191	2015	NWI	North	-90.12475804	41.92109661
1111	2015	NWI	North	-90.125139	41.933221
1213	2015	Focal	North	-90.14648711	42.06819575
1161	2015	NWI	North	-89.49401747	41.63626891
1183	2015	Focal	North	-89.33144697	41.29861612
1203	2015	Focal	North	-89.32316421	41.21503855
1253	2015	Focal	North	-89.31741593	41.1760152
1301	2015	NWI	North	-89.943157	41.410424
181	2015	NWI	North	-89.32167568	41.32169964
21001	2015	NWI	South	-88.83946186	37.25695516
21011	2015	NWI	South	-90.150623	38.660581
21021	2015	NWI	South	-90.0709365	38.63214077
21031	2015	NWI	South	-90.09869841	38.65573437
21041	2015	NWI	South	-90.0944123	38.68779014
21051	2015	NWI	South	-89.8393654	38.59469238
21071	2015	NWI	South	-90.85250555	39.3637713
21081	2015	NWI	South	-87.98300684	38.14771067
21091	2015	NWI	South	-90.20432036	39.70936369
21321	2015	NWI	South	-90.00632671	40.35134837
21343	2015	Focal	South	-89.89898094	40.4523834
21353	2015	Focal	South	-88.70800368	39.62115689
21383	2015	Focal	South	-89.21734897	38.8121772
21403	2015	Focal	South	-90.94195231	39.45324213
21413	2015	Focal	South	-89.87152314	40.52365457
21423	2015	Focal	South	-90.52793995	38.93994097
1121	2015	NWI	North	-88.39294919	42.22233117
1141	2015	NWI	North	-88.13707523	41.33680624
121	2015	NWI	North	-88.29699048	42.22474515
1243	2015	Focal	North	-89.44031344	40.9131159
1312	2015	CTAP	North	-88.36830505	41.9993645
1312	2015	CTAP	North	-88.37147724	42.06699908
1332	2015	CTAP	North	-87.93413215	41.58285735
1362	2015	CTAP	North	-88.00664672	41.82618499
1372	2015	CTAP	North	-88.24342949	41.83355857
141	2015	NWI	North	-88.20377299	42.30677784

Site Number	Year	Category	Region	Longitude	Latitude
1412	2015	CTAP	North	-88.66210886	41.53938174
1422	2015	CTAP	North	-88.31784197	42.06101775
171	2015	NWI	North	-88.30183991	41.37571335
1101	2015	NWI	North	-90.08985822	40.31398399
1223	2015	Focal	North	-89.43363245	40.92686476
1233	2015	Focal	North	-89.40829104	40.93997625
1283	2015	Focal	North	-89.4297148	41.02521392
21393	2015	Focal	South	-88.65388512	40.22803873
22031	2015	NWI	South	-90.05664399	40.34875984
21393	2015	Focal	South	-88.68850677	40.22286353
13013	2016	Focal	North	-89.32735746	41.21210385
23113	2016	Focal	South	-90.06540896	40.34169045
23123	2016	Focal	South	-89.88028069	40.51506559
23133	2016	Focal	South	-89.21210481	38.81569393
23143	2016	Focal	South	-90.93835676	39.45241904
23153	2016	Focal	South	-90.51780708	38.93973761
23163	2016	Focal	South	-89.14523347	37.28800041
23173	2016	Focal	South	-88.68840915	40.22247057
23183	2016	Focal	South	-89.99041187	40.33898345
23193	2016	Focal	South	-89.90132217	40.44554715
23213	2016	Focal	South	-88.2888956	37.66750088
23223	2016	Focal	South	-90.85326297	39.36294218
23233	2016	Focal	South	-90.00692611	40.35138124
13011	2016	NWI	North	-87.74383158	41.53270586
13022	2016	CTAP	North	-89.56679275	42.47792774
13023	2016	Focal	North	-89.4326097	40.93598311
13021	2016	NWI	North	-88.16034815	42.47142531
13033	2016	Focal	North	-91.05822654	40.83321035
13031	2016	NWI	North	-88.48482653	42.31156661
13041	2016	Focal	North	-90.14265601	42.07018024
13042	2016	NWI	North	-90.12541135	41.91061963
13053	2016	Focal	North	-89.43303702	41.02609717
13051	2016	NWI	North	-90.41840014	42.29295227
13063	2016	Focal	North	-89.43104884	40.92807979
13061	2016	NWI	North	-90.06499288	41.63361621
13071	2016	Focal	North	-89.22889829	41.68598622
13071	2016	NWI	North	-91.06646073	41.22963415
13093	2016	Focal	North	-87.888495	42.33222135

Site Number	Year	Category	Region	Longitude	Latitude
13091	2016	NWI	North	-91.00271086	40.90134565
13103	2016	Focal	North	-89.19148004	41.71905869
13101	2016	NWI	North	-90.24506855	41.75144398
13112	2016	CTAP	North	-88.0763995	41.96638224
13111	2016	NWI	North	-88.94457961	41.32354604
13122	2016	CTAP	North	-89.27850378	41.81564728
13121	2016	NWI	North	-90.11822784	41.18193702
13132	2016	CTAP	North	-88.01974164	41.68106514
13131	2016	NWI	North	-90.12069696	42.16438379
13141	2016	NWI	North	-90.37282048	42.26720125
13151	2016	NWI	North	-89.43936324	40.93949191
13161	2016	NWI	North	-90.38185268	41.52078468
23012	2016	CTAP	South	-89.74413306	40.24537807
23011	2016	NWI	South	-89.46667572	40.12047281
23022	2016	CTAP	South	-89.27441406	39.46964635
23021	2016	NWI	South	-90.49515132	39.95996228
23032	2016	CTAP	South	-88.55623378	38.94666389
23031	2016	NWI	South	-90.55939751	40.52083106
23052	2016	NWI	South	-90.54562991	39.97623317
23052	2016	CTAP	South	-87.85035171	38.33616293
23051	2016	NWI	South	-91.30661821	39.76849233
23061	2016	NWI	South	-89.95091681	40.32499339
23072	2016	CTAP	South	-89.31236764	37.57165711
23071	2016	NWI	South	-90.04947586	38.67160249
23081	2016	NWI	South	-88.65560158	37.41207295
23091	2016	NWI	South	-88.57852328	37.70854241
23101	2016	NWI	South	-88.00179932	38.17817151
23112	2016	CTAP	South	-89.35812591	37.42202122
23122	2016	CTAP	South	-89.36861842	37.38401019
301	2017	CTAP	North	-89.46878322	42.47853041
302	2017	CTAP	North	-89.99110588	42.41742969
307	2017	CTAP	North	-89.81459507	42.26534843
392	2017	NWI	North	-88.48529228	42.31097817
304	2017	CTAP	North	-88.37267169	42.33324051
391	2017	NWI	North	-88.0359418	42.2814846
309	2017	CTAP	North	-87.98686274	42.19376028
7031	2017	NWI	North	-89.56132182	42.15599416
353	2017	Focal	North	-90.14541668	42.06996696
310	2017	CTAP	North	-89.5552791	41.99874222
373	2017	NWI	North	-88.78777572	42.00745404

Site Number	Year	Category	Region	Longitude	Latitude
390	2017	NWI	North	-88.18529257	41.95070386
7123	2017	NWI	North	-90.13851891	41.9795463
7186	2017	NWI	North	-90.157691	41.836548
7177	2017	NWI	North	-89.74897468	41.74853597
314	2017	CTAP	North	-89.49188777	41.67960763
7007	2017	NWI	North	-89.375788	41.690099
357	2017	Focal	North	-89.18768596	41.71502039
7036	2017	NWI	North	-90.174769	41.582077
7048	2017	NWI	North	-90.230137	41.558814
313	2017	CTAP	North	-87.93014475	41.75762773
376	2017	NWI	North	-88.14109513	41.55356526
345	2017	Focal	North	-89.32379297	41.2165206
7117	2017	NWI	North	-90.969073	41.421309
318	2017	CTAP	North	-87.70677698	40.80527258
366	2017	Focal	North	-89.43300216	41.02147338
333	2017	Focal	North	-89.43487757	40.935993
317	2017	CTAP	North	-88.66040363	40.89964132
340	2017	Focal	North	-91.0650777	40.83317322
7004	2017	NWI	North	-91.091088	40.678085
7078	2017	NWI	South	-91.38822866	40.53758709
320	2017	CTAP	South	-90.525984	40.252525
338	2017	Focal	South	-88.68733047	40.22319051
7010	2017	NWI	South	-90.42984582	39.99759909
7147	2017	NWI	South	-90.99553004	39.44496056
363	2017	Focal	South	-90.94138033	39.45098905
364	2017	Focal	South	-90.54133424	38.94459128
372	2017	NWI	South	-90.09630231	38.66475339
371	2017	NWI	South	-90.09035369	38.57592824
329	2017	CTAP	South	-89.9529595	38.00719142
370	2017	NWI	South	-89.24395611	37.84790805
368	2017	NWI	South	-88.74010889	37.3883441
369	2017	NWI	South	-88.68413531	37.35746148
375	2017	NWI	South	-88.75545243	38.20288812
374	2017	NWI	South	-88.81504403	38.67350453
336	2017	Focal	South	-89.21587537	38.80993177
324	2017	CTAP	South	-88.58438917	38.99304807
342	2017	Focal	South	-90.0535819	40.35468503
343	2017	Focal	South	-90.006593	40.350037
344	2017	Focal	South	-89.98652472	40.34059829

Site Number	Year	Category	Region	Longitude	Latitude
7003	2017	NWI	South	-89.816287	40.396697
356	2017	Focal	South	-89.955296	40.445451
361	2017	Focal	South	-89.8930285	40.45401161
393	2017	Focal	South	-90.057059	40.45169
335	2017	Focal	South	-89.86195187	40.52649609
